# Feasibility of fast cardiovascular magnetic resonance strain imaging in patients presenting with acute chest pain

**DOI:** 10.1371/journal.pone.0251040

**Published:** 2021-05-03

**Authors:** Johannes H. Riffel, Deborah Siry, Janek Salatzki, Florian Andre, Marco Ochs, Lukas D. Weberling, Evangelos Giannitsis, Hugo A. Katus, Matthias G. Friedrich

**Affiliations:** 1 Department of Cardiology, Angiology and Pneumology, University of Heidelberg, Heidelberg, Germany; 2 DZHK (German Centre for Cardiovascular Research), Partner Site Heidelberg, Berlin, Germany; 3 Departments of Medicine and Diagnostic Radiology, McGill University Health Centre, Montreal, Quebec, Canada; Faculty of Medical Science - State University of Campinas, BRAZIL

## Abstract

**Background:**

Cardiovascular magnetic resonance (CMR) is the current reference standard for the quantitative assessment of ventricular function. Fast Strain-ENCoded (fSENC)-CMR imaging allows for the assessment of myocardial deformation within a single heartbeat. The aim of this pilot study was to identify obstructive coronary artery disease (oCAD) with fSENC-CMR in patients presenting with new onset of chest pain.

**Methods and results:**

In 108 patients presenting with acute chest pain, we performed fSENC-CMR after initial clinical assessment in the emergency department. The final clinical diagnosis, for which cardiology-trained physicians used clinical information, serial high-sensitive Troponin T (hscTnT) values and—if necessary—further diagnostic tests, served as the standard of truth. oCAD was defined as flow-limiting CAD as confirmed by coronary angiography with typical angina or hscTnT dynamics. Diagnoses were divided into three groups: 0: non-cardiac, 1: oCAD, 2: cardiac, non-oCAD. The visual analysis of fSENC bull´s eye maps (blinded to final diagnosis) resulted in a sensitivity of 82% and specificity of 87%, as well as a negative predictive value of 96% for identification of oCAD. Both, global circumferential strain (GCS) and global longitudinal strain (GLS) accurately identified oCAD (area under the curve/AUC: GCS 0.867; GLS 0.874; p<0.0001 for both), outperforming ECG, hscTnT dynamics and EF. Furthermore, the fSENC analysis on a segmental basis revealed that the number of segments with impaired strain was significantly associated with the patient´s final diagnosis (p<0.05 for all comparisons).

**Conclusion:**

In patients with acute chest pain, myocardial strain imaging with fSENC-CMR may serve as a fast and accurate diagnostic tool for ruling out obstructive coronary artery disease.

## Background

Suspected acute coronary syndrome (ACS) is one of the most common causes for emergency department (ED) visits [1]. Patients typically present with either chest discomfort or shortness of breath of unknown etiology. As one of the many etiologies of these symptoms, ACS deserves special attention, as the early verification of an acute obstruction or total occlusion of a coronary artery and an associated ischemic myocardial injury requires rapid therapeutic intervention, i.e. coronary revascularization. An early intervention can salvage the myocardium at risk, while in turn, any delay of this therapy leads to additional irreversible myocardial injury with potentially serious consequences for the patient [2, 3].

Very early sequential changes after the onset of acute ischemia, also referred to as the “ischemic cascade”, represent diagnostic targets: The drop of perfusion, caused by the coronary event is followed by a mismatch of energy supply and demand (oxygen deficit). The energy deficit leads to a loss of diastolic, then systolic function, followed by clinically recognizable abnormalities such as electrocardiogram (ECG) changes, and angina [4].

In patients with suspected acute coronary syndrome, ECG and troponin are used as early diagnostic markers. The use of troponin rise as a marker for acute myocardial cell injury has significantly improved the diagnostic work-up of patients with suspected ACS [5]. It is however not specific for ischemic injury caused by coronary artery disease (CAD), and current high-sensitivity test kits can lead to false positive results [6].

Ventricular function can be assessed by echocardiography. The accuracy however is limited by restricted views and interobserver variability. More recently, contrast agents and strain imaging [7–9] are being used more often to in order to improve sensitivity.

Cardiovascular Magnetic Resonance (CMR) is the accepted reference standard for assessment of ventricular function [10, 11]. Recently, fast Strain-ENCoded (fSENC) CMR imaging was introduced as a single heartbeat acquisition technique for assessing myocardial strain, with demonstrated clinical utility [12, 13].

We hypothesized that fSENC-CMR accurately identifies obstructive coronary artery disease (oCAD) (defined as stenosis >70% of reference vessel diameter) in patients with suspected ACS early after onset of chest pain and inconclusive initial clinical screening (patient presentation, clinical examination, ECG, initial hscTnT level) (accuracy rate >80% compared to standard of reference).

## Material and methods

### Study population

We performed a prospective, single-centre pilot study in patients, who were referred to a chest pain unit for suspected acute coronary syndrome. We enrolled patients with acute chest pain after initial hscTnT results and performed a short fSENC-CMR within 1 hour after patient presentation, prior to 2^nd^ hscTnT results. Patients were closely monitored (ECG, pulse oximetry) and were accompanied by a physician during transport as well as during the CMR scan. Table 1 lists inclusion and exclusion criteria. oCAD was defined as either unstable angina without high-sensitivity cardiac troponin T (hscTnT) dynamics or a significant rise/fall of hscTnT combined with angiographically confirmed obstructive CAD (visually graded diameter stenosis of >70%). CMR was performed after first clinical assessment including patient history, physical examination, ECG and laboratory testing including initial hscTnT level within 1 hour after patient presentation. As a standard of reference, we used the final clinical diagnosis, for which cardiologists on staff primarily used clinical information, ECG findings and serial hscTnT values. A fourth-generation high-sensitivity (hs) cTnT assay was used as described in previous studies (Roche Diagnostics Penzberg, Germany) [14].

**Table 1:** Inclusion and exclusion criteria of the study [28].

**Inclusion criteria**	**Exclusion criteria**
• acute chest pain	• acute ST-elevation myocardial infarction
• HEART score ≤6	• hemodynamic instability
• initial hscTnT 5–52 pg/nl	• cardiogenic shock
• signed informed consent	• mechanical complications of MI
	• systolic heart failure (LVEF<40%)
	• life-threatening arrhythmias
	• history of CAD
	• CMR: non-suitable metallic implants
	• CMR: severe claustrophobia

hscTNT: high-sensitive cardiac troponin T, LVEF: left ventricular ejection fraction, CAD: coronary artery disease MI: myocardial infarction CMR: cardiovascular magnetic resonance.

A significant hscTnT dynamic was defined as a kinetic of ≥5 ng/l within 1 hour according to the 0h/1h algorithm [3]. According to clinical assessment, the patients were either discharged (rule-out) or received further tests such as coronary angiography, echocardiography, coronary CT angiography, standard stress CMR, or stress ECG. Final clinical decision-making was performed without the knowledge of fSENC-CMR results. For calculation of the HEART score 5 components were used (history, electrocardiogram (ECG), age, risk factors and troponin), while for each component 0, 1 or 2 points were given. The HEART score has previously been described in detail [15].

The study was approved by the local ethics committee (Ethikkommission Medizinische Fakultät Heidelberg (S-483/2018)). All participants provided informed written consent.

### CMR protocol

The majority of the study population was scanned in a 1.5T whole-body CMR scanner (Ingenia CX, Philips Medical Systems, Best, The Netherlands). Sixteen patients were scanned in a 3T whole-body scanner (Ingenia, Philips Medical Systems, Best, The Netherlands), thus their mapping data were not included in the analysis. Field strength did not impact fSENC image quality.

The CMR protocol included regular cine steady-state free precession (SSFP) images (Field of view (FOV 140 mm^2^, TE 1.38 ms, TR 2.77 ms, flip angle 60°, pixel size 0.88 x 0.88mm^2^, 35 acquired phases, slice thickness 8 mm), myocardial T1 (FOV 100 mm^2^, TE 1.06 ms, TR 2.31ms, flip angle 35°, pixel size 1.17 x 1.17 mm^2^, slice thickness 10 mm) and T2 mapping(FOV 100 mm^2^, TE 24.93 ms, TR 674.16ms, flip angle 90°, pixel size 0.94 x 0.94mm^2^, slice thickness 10 mm) as well as fSENC acquisitions (FOV 100 mm^2^, TE 0.71 ms, TR 12.16 ms, flip angle 30°, pixel size 1 x 1mm^2^, slice thickness 10 mm). Cine SSFP images were obtained in short axis views covering the whole left ventricle (gap 2 mm) as well as in 2-, 3- and 4-chamber views. T1 and T2 maps were analyzed in an apical-midventricular and midventricular-basal short axis view and global values were calculated (R^2^<0.07; endocardial and epicardial offset 10%, number of segments 6) [16].

### Strain analysis

fSENC images were semi-automatically analyzed by a reader certified in the use of the software Myostrain^™^ (Myocardial Solutions, Morrisville, NC, USA) and blinded to laboratory data. After manual contouring in end-systole in 6 slices, segmental and global strain data were automatically calculated and displayed in bull’s eye maps.

**Fig 1:**
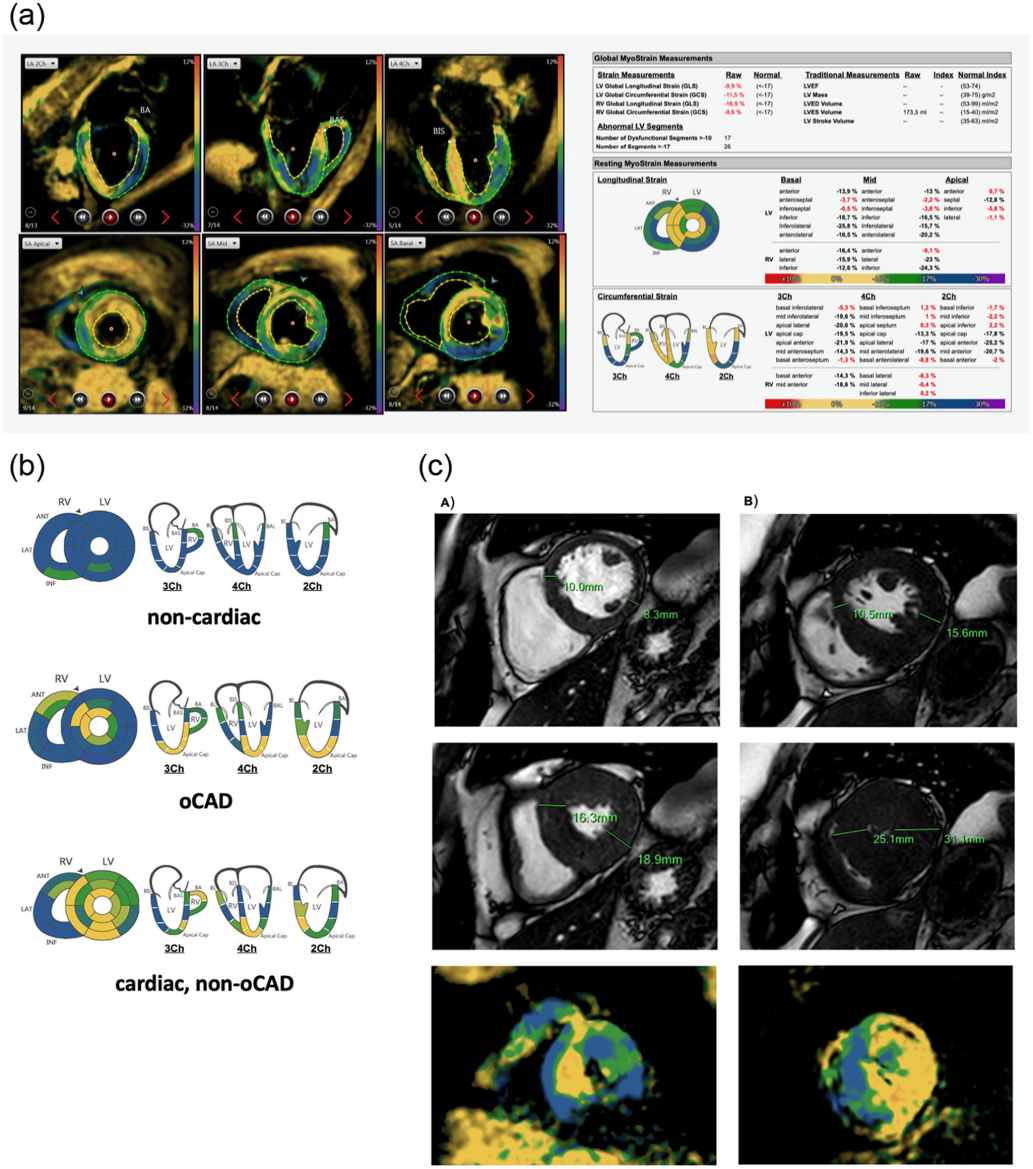
a. Representative example of fSENC manual contouring in endsystole and final report. Left 6 images in clockwise direction: 2 chamber, 3 chamber, 4 chamber view (long-axes for circumferential strain calculation), short basal, mid and apical view (short axes for longitudinal strain calculation). Right image: final report after contouring with global and segmental strain results. b. Representative example of fSENC bull’s-eye maps of longitudinal strain data (left) and circumferential data (right). Color code: blue represents normal contractility (strain<-17), green represents slightly reduced contractility (-10> strain >-17) and yellow depicts dysfunctional segments (strain>-10). Colors vary according to the exact segmental strain value. c. Differentiation between multi-vessel disease and cardiac, non-oCAD using cine and fSENC raw images. A: oCAD—multi-vessel disease 1. cine end-diastole, 2. cine end-systole, 3. fSENC in end-systole/ B: cardiac, non-oCAD pathology 1. cine end-diastole, 2. cine end-systole, 3. fSENC in end-systole.

Global longitudinal strain (GLS) and global circumferential strain (GCS) were calculated by averaging the results from 16 (short axis apical/mid/basal) and 21 (long axis 2CH/3CH/4CH) left-ventricular segments, respectively [17] (Fig 1a).

Using the visual information of the fSENC bull’s-eye maps, patients were triaged into three groups: 0: non-cardiac, 1: oCAD, 2: cardiac, non-oCAD. Classification was based on the independent visual assessment of two different readers. Segments with a strain > -10 were considered dysfunctional, strain > -17 as mildly dysfunctional [18]. Maps devoid of dysfunctional segments were classified to group 0, regional dysfunction was classified to group 1 and maps with global dysfunction incompatible with coronary artery territories were filed to group 2. In order to differentiate multi-vessel disease with larger regional dysfunction from an underlying global cardiac disease, the raw fSENC images were further analyzed and the LV septal and lateral wall thickness was measured. Additionally, the visual distribution of dysfunctional deformation was taken into account. A representative example of each group is given **in** Fig 1b **with visual differentiation between group 1 and 2 further displayed in** Fig 1c.

### Statistics

Quantitative data were presented as mean values ± standard deviation (SD) or as median and interquartile range (IQR).

A sample size of 108 patients was calculated based on our primary endpoint: H0: diagnostic accuracy (fSENC) <80% as compared to the standard of reference (diagnostic accuracy ≥ 90%; power 80%; p-value <5%).

McNemar’s test was applied to register a significant deviation of fSENC-based diagnoses from the reference standard. As GLS and GCS values were not normally distributed (Kolmogorov-Smirnov test), a Mann-Whitney U test was performed to assess for differences between the groups. Receiver Operating Characteristic (ROC) curves were calculated and the area under the curve (AUC) was determined. ROC curves and logistic regression curves were compared using a Hanley and McNeil test [19]. Data of strain differences on a segmental level were not normally distributed (Kolmogorv-Smirnov test) and analyzed using a Mann-Whitney U test and displayed in boxplots. Interobserver reliability was assessed using intraclass correlation coefficient (ICC). P-values <0.05 were regarded as statistically significant. All statistical analyses were performed using the computer programs Excel (Microsoft, Redmond, USA), SPSS (Version 24, IBM, Armonk, USA) and MedCalc (Version 19.2, MedCalc Software, Ostend, Belgium).

## Results

### Patient characteristics

We enrolled 108 patients, of whom 85 were finally diagnosed with non-cardiac chest pain, 6 with cardiac disease but not oCAD (n = 3 Hypertrophic Cardiomyopathy, n = 1 Dilated Cardiomyopathy, n = 1 Myocarditis, n = 1 Pulmonary Hypertension), while 17 patients had a final diagnosis of oCAD, 8 of whom had non-ST-elevation myocardial infarction (NSTEMI), i.e. positive troponin but no ST elevation [3]. Table 2 shows the patient characteristics. Gender was evenly distributed (49 female participants). The mean age was 57±17 years. The average patient presented with two cardiovascular risk factors of which the most common was arterial hypertension (54%). Table 3 depicts patient diagnosis of the 25 patients who underwent coronary angiography.

**Table 2:** Patient characteristics.

		count	mean (± SD)	max/min	median	interquartile range
**Sex**	female	49 (45%)				
	male	59 (55%)				
**Age**			57±17	85/20		
**BMI**			26.6±5.3	52.9/14.8		
**BP (systolic)**			155±20	204/110		
**HR**			71±16	133/32		
**HEART score**	low		41			
	intermediate		67			
**NYHA**	1	74 (69%)				
	2	18 (17%)				
	3	15 (14%)				
	4	1 (1%)				
**EF**			65.4±12.9	96.0/20.5	66.4	16.5
**EDV**			92.8±39.4	237.2/40.3	79.1	52.5
**ESV**			32.5±21.5	132.6/4.5	26.5	18.8
**cvRF**			2	5/0		
**Diabetes**		9 (8%)				
**Hypertension**		58 (54%)				
**Hypercholesterinemia**		34 (31%)				
**Familial predisposition**		31 (29%)				
**nicotine (py)**	non-smoker	62 (57%)	0±1	9/0		
	past smoker	32 (30%)	19±15	45/1		
	smoker	14 (13%)	25±20	60/3		
**Chest pain duration (h) (oCAD)**			15.6	>24/1	24	21
**Chest pain duration (h) (cardiac, non-oCAD)**			15	>24/0.5	22	20
**Chest pain duration (h) (non-cardiac)**			14.9	>24/1	16	19
**hscTnT 0h**			11±8	49/5		
**hscTnT 1h**			15±21	112/3		
**Δ hscTnT (kinetics)**			5±18	+93/-28		
**Diagnostic procedures**	stress ECG	7 (6%)				
	echocardiography	6 (6%)				
	standard CMR	1 (1%)				
	CT angiography	1 (1%)				
	coronary angiography	25 (23%)				

max: maximum, min: minimum, SD: standard deviation BMI: body mass index, BP: blood pressure, HR: heart rate, NYHA: New York Heart Association, EF: ejection fraction, ESV: End-systolic volume, EDV: End-diastolic volume, cvRF: cardiovascular risk factors, py: pack years, h: hours, oCAD: obstructive coronary artery disease, hscTNT: high-sensitive cardiac troponin T, ECG: electrocardiogram, CMR: cardiovascular magnetic resonance, CT: computed tomography.

**Table 3:** fSENC and hscTnT results of the 25 patients who underwent coronary angiography according to patient diagnosis (oCAD, cardiac, non-oCAD and non-cardiac).

	oCAD	cardiac, non-oCAD	non-cardiac
coronary angiogram +	**17**	**0**	**0**
coronary angiogram -	**0**	**3**	**5**
fSENC +	**14**	**0**	**0**
fSENC -	**3**	**3**	**5**
hscTnT +	**8**	**1**	**2**
hscTnT -	**0**	**1**	**3**
Observe	**7**	**1**	**2**

oCAD: obstructive coronary artery disease, hscTNT: high-sensitive cardiac troponin T.

### fSENC CMR triage results

**Fig 2:**
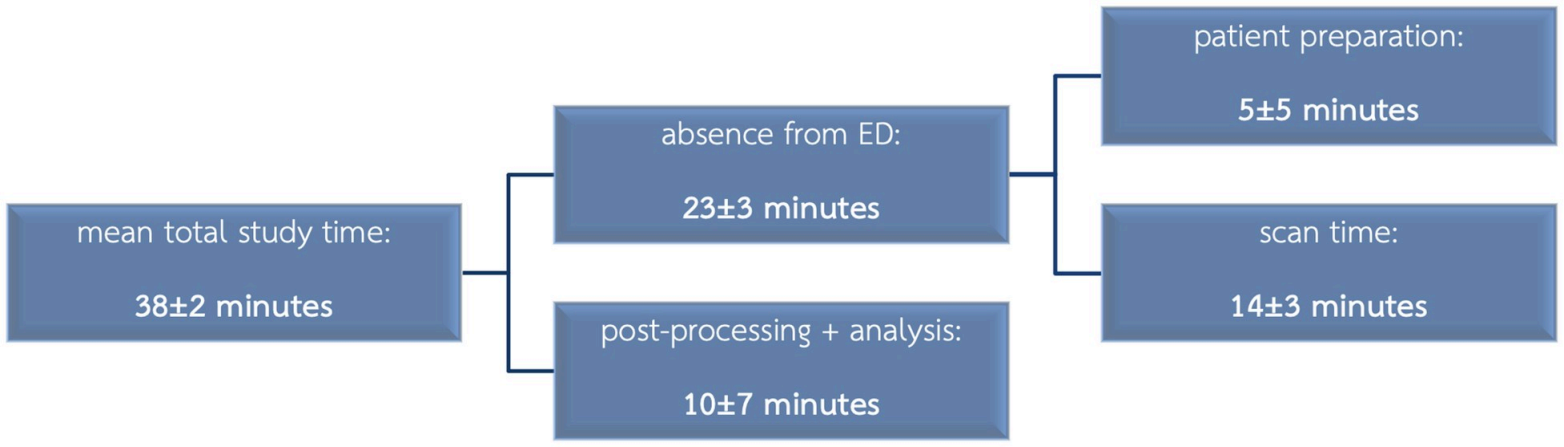
Study duration timeline.

All patients completed the CMR protocol as planned. Mean CMR image acquisition time was 14±3 minutes (median: 16, IQR: 5). Average total study time from arrival in the MR suite to end of image analysis was 38±2 minutes. This includes patient preparation within the MR suite (5±5 minutes), image generation (14±3 minutes), post-processing and analysis (10±7 minutes). Patients were absent from the ED for about 23±3 minutes (Fig 2).

Using the visual information of the fSENC bull’s-eye maps, patients were triaged into three groups as described above. Based on the pre-defined criteria, 68 patients were classified to group 0 (non-cardiac), 26 patients were classified to group 1 (oCAD) and 14 patients were classified to group 2 (cardiac, non-oCAD). There were 12 false positive results and 3 false negative reports in predicting oCAD (Table 4), resulting in a sensitivity of 82.4%, specificity of 86.8% as well as positive predictive value of 53.8% and negative predictive value of 96.3%. The diagnostic accuracy was 86.1%. Of note, 8 patients underwent additional coronary angiogram and 7 patients performed an additional stress ECG in order to rule-out oCAD during routine diagnostic work-up. All these 15 patients had a negative fSENC-CMR result prior to coronary angiography and stress ECG.

**Table 4:** Classification with visual analysis of fSENC (fSENC + means classification as oCAD (1), fSENC—Means classification as non-cardiac (0) or cardiac; non-oCAD (2)).

		fSENC +	fSENC -	
oCAD (1)		14	3	17
non-cardiac (0)/ cardiac, non-oCAD (2)	0	12	65	77
	2	0	14	14
	0 + 2	12	79	91
		26	82	108

oCAD: obstructive coronary artery disease, hscTNT: high-sensitive cardiac troponin T

### ROC curves

**Fig 3:**
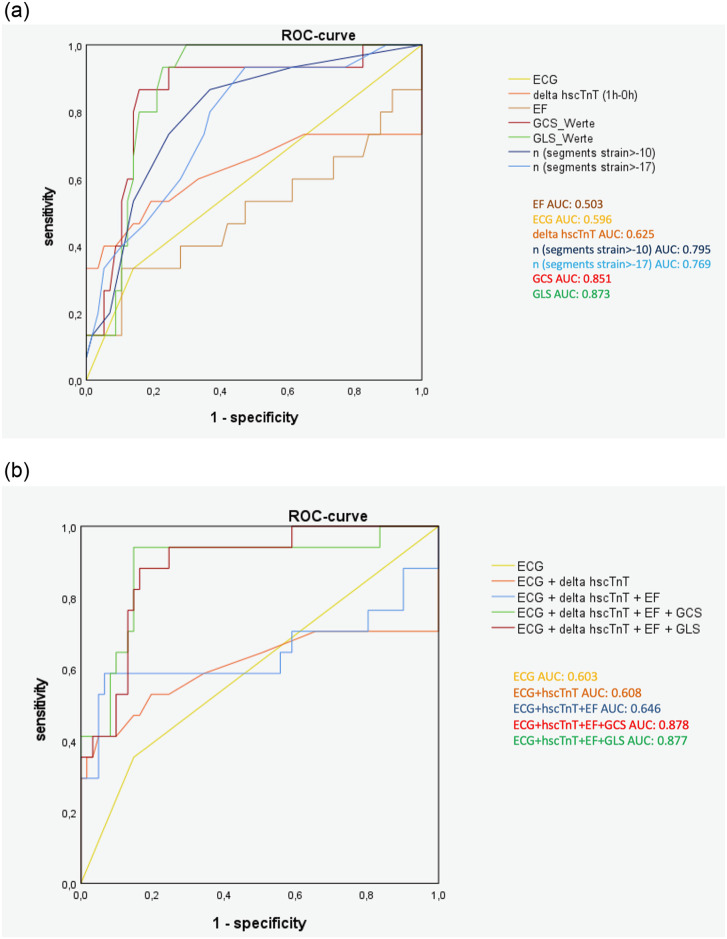
a Comparison of ROC curves: Single diagnostic methods. The area under the curve (AUC) for GCS and GLS was 0.851 and 0.873 respectively. Both values were statistically significant (p<0.0001). GCS and GLS performed significantly better than ECG (AUC: 0.596), hscTnT (AUC:0.625) and EF (AUC: 0.503) (GCS vs. ECG: p<0.015; GCS vs. hscTnT: p<0.05; GLS vs. ECG: p<0.004; GLS vs. hscTnT: p<0.035; GCS vs. EF: p<0.0015; GLS vs. EF: p<0.0005). The AUC for the number of segments with a strain >-17 and >-10 was 0.769 (p<0.0001) and 0.795 (p<0.001), respectively. Segmental strain performed significantly better than ECG and EF (segments strain>-17 vs. ECG: p = 0.06; segments strain >-17 vs. hscTnT: p = 0.22; segments strain>-17 vs. EF: p<0.03; segments strain>-10 vs. ECG: p<0.05; segments strain>-10 vs. hscTnT: p = 0.16; segments strain>-10 vs. EF: p<0.01;) but was inferior to global strain (GCS vs. segments strain>-17: p = 0.25; GLS vs. segments strain>-17: p = 0.11; GCS vs. segments strain>-10: p = 0.34; GCS vs. segments strain>-17: p = 0.25). b Logistic regression analysis: Combined diagnostic methods. The area under the curve (AUC) for GCS and GLS in combination with routine clinical diagnostic work-up results (ECG + hscTnT kinetics + EF) was 0.878 and 0.877, respectively. Both values were statistically significant (p<0.0001). GCS and GLS performed significantly better than ECG, hscTnT kinetics and EF (AUC: 0.646) (ECG+hscTnT+EF vs. ECG+hscTnT+EF+GCS: p<0.008; ECG+hscTnT+EF vs. ECG+hscTnT+EF+GLS: p<0.01).

Both, GCS and GLS accurately identified oCAD. The area under the curve (AUC) for GCS and GLS was 0.851 (95% CI: 0.74–0.97) and 0.873 (95% CI: 0.79–0.95), respectively. Both values were statistically significant (p<0.0001). The optimum threshold for GCS was at -18.25 (sensitivity 94.1%, specificity 75.4%) and -18.75 for GLS (sensitivity 94.1%, specificity 72.1%; Fig 3a and 3b). AUC values were lower for ECG, hscTnT dynamics and EF (ECG: 0.596, p = 0.253; delta hscTnT: 0.625, p = 0.138; EF: 0.503, p = 0.972). Both, GCS and GLS performed significantly better than ECG, delta hscTnT and EF (GCS vs. ECG: p<0.015; GCS vs. delta hscTnT: p<0.05; GLS vs. ECG: p<0.004; GLS vs. delta hscTnT: p<0.05; GCS vs. EF: p<0.0015; GLS vs. EF: p<0.0005). When added to routine clinical diagnostic work-up (ECG, hscTnT kinetics and EF), both GCS and GLS had a higher AUC (AUC ECG+hscTnT: 0.608, p = 0.175; AUC ECG+hscTnT+EF: 0.646, p = 0.067; AUC ECG+hscTnT+EF+GCS: 0.878, p = 0.0001; AUC ECG+hscTnT+EF+GLS: 0.877, p = 0.0001) with a significantly better performance than combined ECG, hscTnT kinetics and EF (ECG+hscTnT+EF vs. ECG+hscTnT+EF+GCS: p<0.008; ECG+hscTnT+EF vs. ECG+hscTnT+EF+GLS: p<0.01).

The number of segments with strain >-17 and strain >-10 resulted in an AUC of 0.769 (95% CI: 0.64–0.90; p<0.001) and 0.795 (95% CI: 0.67–0.92; p<0.0001)), respectively. The optimal threshold for the number of segments with strain >-17 was n = 7.5 (sensitivity 80.0%, specificity 63.2%) whilst for the number of segments with strain >-10 n = 1.5 was optimal (sensitivity 86.7%, specificity 63.2%). The segmental analysis proved to be beneficial in a routine clinical diagnostic work-up setting (segments strain >-17 vs. ECG, p = 0.06; segments strain >-10 vs. ECG, p<0.05; segments strain >-17 vs. delta hscTnT, p = 0.22; segments strain >-10 vs. delta hscTnT, p = 0.16; segments strain >-17 vs. EF, p; segments strain >-10 vs. EF, p<0.01), however inferior to global strain values for oCAD identification (GCS vs. segments strain >-17, p = 0.25; GCS vs. segments strain >-10, p = 0.34; GLS vs. segments strain >-17, p = 0.11; GLS vs. segments strain >-10, p = 0.18).

### Global strain

**Fig 4:**
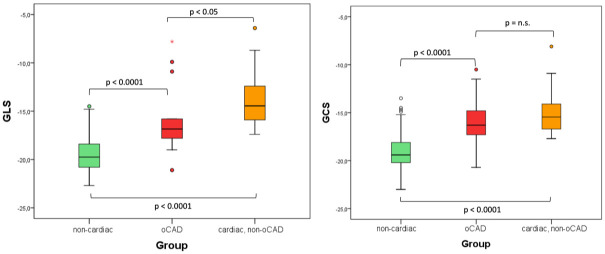
Nomogram. Nomogram for low (0.9–1.7% risk of MACE) and intermediate (12–16.6% risk of MACE) HEART score: Pre-Test Probability according to HEART score, Post-Test Probability as a combined diagnostic input of the HEART score and fSENC results (LR+ 6.24/ LR– 0.2). Accordingly, post-Test probability of low and intermediate HEART score with negative fSENC results was 0.2% and 3.5% respectively, whereas for positive fSENC results a 9% and 50% post-test probability could be assessed.

Furthermore, both GCS and GLS differed significantly between the non-cardiac group and the cardiac group (oCAD vs cardiac, non-oCAD; p<0.0001). Of note, while GLS was significantly different in all patient groups, GCS was unable to distinguish between oCAD and cardiac, non-oCAD cases (Fig 4a and 4b).

**Fig 5:**
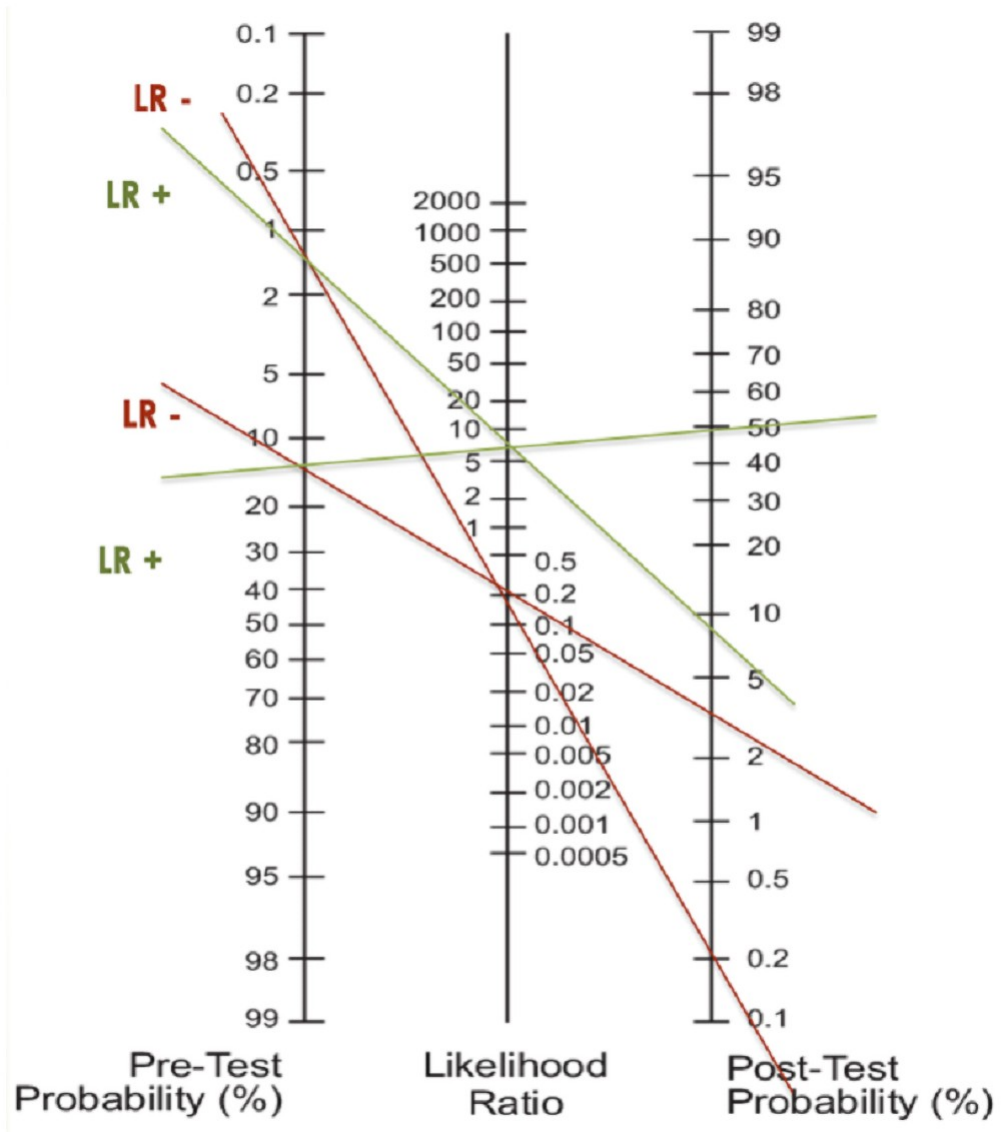
a and b Boxplot of GCS (3a) and GLS (3b) values in the fSENC triage groups 0) non-cardiac 1) oCAD 2) cardiac, non-oCAD. GCS was significantly better for differentiation between the non-cardiac group and the oCAD group, while there was no significant difference between the oCAD group and cardiac, non-oCAD group. Values for GLS differed significantly between all groups.

In order to assess the incremental diagnostic benefit of fSENC to established risk scores, the likelihood ratio (LR) for fSENC and the HEART score was calculated, resulting in a positive LR of 6.24, and a negative LR of 0.2. When used for clinical decision-making, patients with low or moderate pre-test probability and negative fSENC results could have been safely discharged (0.2% MACE risk for low pre-test probability and 3.5% for moderate pre-test variability), while patients with abnormal fSENC results should be further examined (9% MACE risk for a pre-test probability, 50% MACE risk for moderate pre-test probability) (Fig 5).

### Segmental strain

We performed a separate analysis regarding the number of dysfunctional segments (strain >-10) and number of mildly/marginally dysfunctional + dysfunctional segments (strain >-17) within the three patient groups.

**Fig 6:**
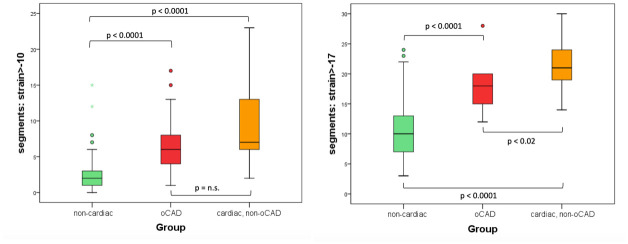
a and b Boxplot of number of dysfunctional segments in the three patient groups (4a: strain >-10 4b: strain >-17) based on 16 short axis segments and 21 long axis LV-segments. The number of severely dysfunctional segments was significantly different between the non-cardiac and oCAD group, differences between the oCAD group and cardiac, non- oCAD group were not significant. The number of slightly dysfunctional segments was a significant parameter for differentiation between all three patient groups.

Especially, the number of segments with strain values > -17 was an excellent parameter for differentiation between the patients’ final diagnoses (Fig 6a and 6b).

### Mapping

Global as well as regional T1 and T2 values did not show significant difference between the three patient groups. We performed a subgroup analysis of the 17 oCAD patients. Regional T1, T2 and GLS, GCS values were calculated according to the culprit coronary lesions. We noted however a significant correlation between regional circumferential strain (RCS) and regional T2 (Pearson’s 0.776, p<0.001).

### Interobserver reliability

15 patients were separately contoured and analyzed by a second certified reader, blinded to the results of the other reader. The resulting intraclass correlation coefficient (ICC) was 0.91 and 0.98 for GLS and GCS, respectively (p<0.0001). For the number of segments >-10 and >-17, the ICC was 0.96 and 0.96, respectively (p<0.0001).

## Discussion

To our knowledge, this is the first study to evaluate strain measurements using CMR for identifying obstructive CAD in patients with acute chest pain.

Although CMR is the reference standard for assessment of cardiac function [11] and plays a key role in detecting ischemia in patients with suspected CAD [20], CMR has not been integrated in the diagnostic work-up and risk stratification in patients with acute chest pain mainly due to lengthy protocols, costs and limited access to MRI systems [21, 22].

fSENC acquires all necessary data for one image slice within one single heartbeat. This allows for a very fast quantitative assessment of myocardial deformation, while not being dependent on long breath-holds [13, 23].

### Visual analysis of fSENC

The main finding of our pilot study was that we were able to safely triage patients (0: no cardiac cause/ 1: oCAD/ 2: cardiac, non-oCAD) according to a simple visual analysis of fSENC Bull´s Eye maps.

Our results indicate that in patients presenting with acute chest pain, no history of significant CAD, and inconclusive initial clinical screening, fSENC-CMR can identify oCAD by detecting associated subtle wall motion abnormalities without pharmacological or other interventions.

Particularly, the high sensitivity and negative predictive value need to be highlighted. Negative fSENC-CMR results may allow for a safe discharge, rendering additional diagnostic procedures such as coronary angiography or stress ECG according to ED protocol unnecessary.

Of note, the positive predictive value was relatively low. As the sample size was relatively small and in particular the number of patients with confirmed oCAD was low, the positive predictive value may be significantly higher in a larger patient population.

### Quantification of fSENC

Besides the visual analysis, quantifiable markers like GLS, GCS and the number of segments with increased strain yielded a high diagnostic accuracy in identifying patients with oCAD. Here, the most sensitive and specific strain marker for identifying oCAD was GCS. These findings are consistent with previously published echocardiographic data [9] and extend our knowledge about fSENC in patients with CAD. In a study in patients 3 days after successful coronary revascularization after acute myocardial infarction, SENC-CMR rapidly measured LV function and discriminated transmural from non-transmural infarcts [18]. In particular, circumferential strain was very useful, consistent with the results of our study. Although GLS had a marginally lower sensitivity and specificity than GCS for oCAD identification, it was the best marker to further distinguish between oCAD and cardiac, non-oCAD disease. This suggests that, whilst GCS detects myocardial injury relatively well, GLS is the more susceptible parameter when it comes to differentiating oCAD from cardiac disease of other causes.

Interestingly, the ROC and logistic regression analysis revealed that GCS and GLS not only performed significantly better than stand-alone ECG, hscTnT kinetics, or EF but also were beneficial when added to routine diagnostic work-up.

Irrespective of ECG and biomarkers, addition of fSENC was significantly beneficial when added to a theoretical model, which uses the HEART Score.

### Segmental strain analysis

Furthermore, we analyzed myocardial deformation on a segmental basis and found that the number of segments with strain values >-17 correctly predicted the diagnostic group of the patients. Here, we found that patients with oCAD showed a smaller number of dysfunctional segments than patients referred to the cardiac, non-oCAD group, who mainly showed global deformation abnormalities. However, ROC curves revealed that a segmental strain analysis was inferior to global strain for oCAD identification while still surpassing routine clinical diagnostic work-up (ECG, hscTnT kinetics and EF). This may be due to the fact that in the oCAD group only few segments were affected but resulted in a strongly reduced overall myocardial contractility. Therefore, global strain may be the better early marker to identify oCAD while the number of affected segments might increase later.

Interestingly, 8 of the 14 patients with global myocardial deformation abnormalities—indicating an underlying cardiac disease other than oCAD—were finally diagnosed with non-cardiac chest pain and therefore discharged whereas in the other 6 patients underlying cardiac disease was diagnosed during work-up in the ED. This indicates that in 8 patients, who were discharged after clinical assessment, ECG and laboratory testing, the underlying heart disease was missed without fSENC-CMR. Therefore, fSENC-CMR may allow for earlier identification of underlying cardiac pathologies other than CAD.

In 2015 Chu et al. performed CMR in 32 patients presenting with ACS and unobstructed coronary arteries and found that in most cases CMR correctly identified the underlying aetiology. In contrast to our study, the anatomy of coronary arteries was known as CMR was conducted 2–4 h after negative cardiac catheterization, while scanning time was longer (42.5 ± 18.2 min) [24].

In 2003, Kwong et al. analyzed the diagnostic performance of CMR in patients with ACS. In this study CMR was performed in 161 patients with chest pain and no ST elevation within 12 hours after presentation including perfusion, LV function and LGE imaging. This combined protocol had a sensitivity of 84% and a specificity of 85% for identifying ACS. However, a major drawback was the prolonged scan time resulting in a patient absence from the ED of 58 ± 10 minutes [21].

We performed CMR early after clinical assessment (including ECG and laboratory testing) and were able to complete the scan in an average time of less than 15 minutes with a (closely monitored) absence from the ED of around 23±3 minutes, resulting in a comparable diagnostic accuracy (sensitivity of 82.4%, specificity of 86.8%).

We are aware that CMR is not widely available in emergency and interventional cardiology departments. However, as CMR is moving towards short, focused scan protocols and is increasingly recommended in recent guidelines [25], we believe that this modality will be more widespread in future and also be available for patients in the ED.

Besides saving critical time in certain patients (intermediate pre-test probability), another advantage of fSENC-CMR compared to a “traditional” CMR protocol including first-pass perfusion and LGE is that it is completely free of pharmaceutical stress agents or contrast agents. fSENC CMR may serve as a needle-free diagnostic tool to triage patients with acute chest pain and intermediate risk prior to the hscTnT results. In future, this may help to discharge patients early and safely thereby avoiding unnecessary invasive procedures and consequently save hospital expenses.

### Comparison with Feature Tracking and other modalities

While cine-based strain analysis such as Feature Tracking (FT) imaging may further reduce scan time by obviating the need to acquire additional sequences to determine strain, it is more susceptible to through-plane motion artifacts and partial volume effects. Furthermore, FT is limited by pixel size [26]. On the other hand, cardiac computed tomography (CT) is able to visualize coronary anatomy and has high predictive performance for outcome in patients with chest pain [27]. However, CT requires radiation and contrast agents and does not provide information about myocardial tissue composition or dynamic vascular function.

Compared to echocardiographic assessed strain imaging—which has to be regarded as a valid alternative modality to assess deformation in an emergency department—CMR is less dependent on image quality and beyond that offers additional information about fibrosis and edema.

### Mapping

Interestingly, global and regional T1 and T2 Mapping failed to differentiate between the three groups in our study.

However, in oCAD patients regional T2 values displayed a significant correlation with regional circumferential strain. A potential explanation may be the detection of an early subendocardial edema with T2 Mapping in this patient group. However, CMR may have been performed too early to detect injury with T1 Mapping or allow for distinguishing between the different patient diagnoses. Whether T2 mapping combined with fSENC may help in differentiating patients with acute ischemia from those with preexisting scar has to be addressed in subsequent studies.

## Limitations

This is the first study to examine the efficacy of fSENC in an acute setting—findings need to be verified in studies with larger sample sizes in order to determine cutoff values which could further increase the positive predictive value. The final sample size of those deemed to have oCAD is small and therefore results should at this point be interpreted with caution and require validation in a large randomized setting.

Patients with history of CAD were excluded from our study in order to reduce probability of pre-existing myocardial scar. Therefore, our findings need to be confirmed in studies with larger populations, including patients with varying clinical context, e.g. history of CAD or systolic heart failure. As CAD is a frequent condition in an all-comers population, differentiation between novel onset of ischemia and pre-existing scar is very important and needs to be evaluated in further studies. Furthermore, severe three vessel disease may mimic cardiomyopathies due to global impairment of LV function which may not be differentiated with fSENC. The classification to the three patient groups was based on visual assessment by two individual readers and was therefore user-dependent.

The pretest probability in our population for CAD was low to intermediate, which explains the low number of further diagnostic test or coronary angiography. The majority of patients included in our study did not receive further coronary imaging. oCAD was ruled out based on fSENC alone while clinical evaluation, biomarkers and ECG served as standard of truth. Our results may not be applied to populations of high risk for CAD. Furthermore, Fractional Flow Reserve (FFR) was not performed in patients who received coronary angiography. Significant CAD was determined by visual assessment of the examiner. Of, note, no extended follow-up was performed which might enable evaluation of cardiovascular risk.

T2 and T1 mapping failed to find significant differences between the three groups, which may be due to the low number of patients with oCAD and low to intermediate pre-test probability for coronary artery disease in our population. Mapping may be a more relevant factor in a study population with more patients with higher risk for NSTEMI or oCAD.

To perform CMR in patients with chest pain and strain imaging as assessed in this study, a suitable infrastructure and profound expertise is inevitable and even more important than in “regular” CMR examinations as results have to be interpreted more rapidly and patient safety is *the* crucial factor. We are aware that CMR is still not widely available in emergency departments or hospitals in general and that our protocol may be difficult to apply in smaller or less experienced hospitals.

Furthermore, we did not compare fSENC CMR with echocardiographic assessed strain imaging in our study.

Additionally, we did not apply stress testing as fSENC was acquired at rest only. Given the higher risk for adverse events when using stress protocols, their use may only be justified if associated with a significantly higher diagnostic accuracy.

## Conclusions

Our pilot data indicate that in patients presenting with acute chest pain and inconclusive initial clinical screening, fast CMR strain imaging using fSENC can be used to rule out obstructive coronary artery disease. The fast CMR protocol appears feasible and safe. If confirmed, fast CMR strain imaging could be integrated into efficient clinical workflows.

## Abbreviations

ACS: Acute coronary syndrome
AUC: Area under the curve
CMR: Cardiovascular magnetic resonance
ECG: Electrocardiogram
ED: Emergency department
EDV: End-diastolic volume
EF: Ejection fraction
ESV: End-systolic volume
fSENC: fast Strain ENCoded
GCS: Global circumferential strain
GLS: Global longitudinal strain
hscTnT: High-sensitive Troponin T
ICC: Intraclass correlation coefficient
LR: Likelihood ratio
oCAD: Obstructive coronary artery disease
RCS: Regional circumferential strain
ROC: Receiver Operating Characteristic
